# The genome sequence of the Dot Moth, *Melanchra persicariae* (Linnaeus, 1761)

**DOI:** 10.12688/wellcomeopenres.19410.1

**Published:** 2023-04-27

**Authors:** Douglas Boyes, Peter W.H. Holland, University of Oxford and Wytham Woods Genome Acquisition Lab, Darwin Tree of Life Barcoding collective, Wellcome Sanger Institute Tree of Life programme, Wellcome Sanger Institute Scientific Operations: DNA Pipelines collective, Tree of Life Core Informatics collective, Darwin Tree of Life Consortium

**Affiliations:** 1 UK Centre for Ecology & Hydrology, Wallingford, England, UK; 2 University of Oxford, Oxford, England, UK

**Keywords:** Melanchra persicariae, Dot Moth, genome sequence, chromosomal, Lepidoptera

## Abstract

We present a genome assembly from an individual male *Melanchra persicariae* (the Dot Moth; Arthropoda; Insecta; Lepidoptera; Noctuidae). The genome sequence is 647.9 megabases in span. Most of the assembly is scaffolded into 31 chromosomal pseudomolecules, including the Z sex chromosome. The mitochondrial genome has also been assembled and is 15.4 kilobases in length.

## Species taxonomy

Eukaryota; Metazoa; Ecdysozoa; Arthropoda; Hexapoda; Insecta; Pterygota; Neoptera; Endopterygota; Lepidoptera; Glossata; Ditrysia; Noctuoidea; Noctuidae; Hadeninae; *Melanchra*; *Melanchra persicariae* (Linnaeus, 1761) (NCBI:txid987979).

## Background

The Dot Moth, *Melanchra persicariae,* is an easily recognised member of the family Noctuidae. The typical form of the moth has almost uniformly blue-black forewings and a bright white reniform stigma (kidney mark), giving the moth its common name. The larva may be green or brown, but always has distinctive markings with three short parallel cream stripes just behind the head and a series of forward-pointing chevron marks on each segment meeting to form triangles when viewed dorsally ( Stokoe, 1948). The larvae feed at night and day on a wide variety of herbaceous plants including nettle, dock and bindweeds, or the foliage of deciduous shrubs and trees. The adult moth has a summer flight period, peaking in July in Britain and Ireland ( Randle *et al.*, 2019). The larva feeds through the autumn months before overwintering as a pupa.

*M. persicariae* can be found in woodland, hedgerows, waste ground and garden habitats, and has been recorded across much of Europe and east across Eurasia to Japan ( GBIF Secretariat, 2022). In the UK, the moth can be locally common in parts of southern England and Wales, although it has declined in abundance over the last 50 years ( Randle *et al.*, 2019). This moth is scarce in Scotland and is considered ‘very rare’ in Northern Ireland ( NBN Atlas Partnership, 2022; Randle *et al.*, 2019; Thompson & Nelson, 2003). The species has a patchy distribution in Ireland, with most records from coastal suburban areas ( MothsIreland, 2022).

A genome sequence for *M. persicariae* will facilitate studies into molecular adaptations to polyphagy and contribute to a growing dataset of resources for understanding lepidopteran biology.

## Genome sequence report

**Figure 1.**
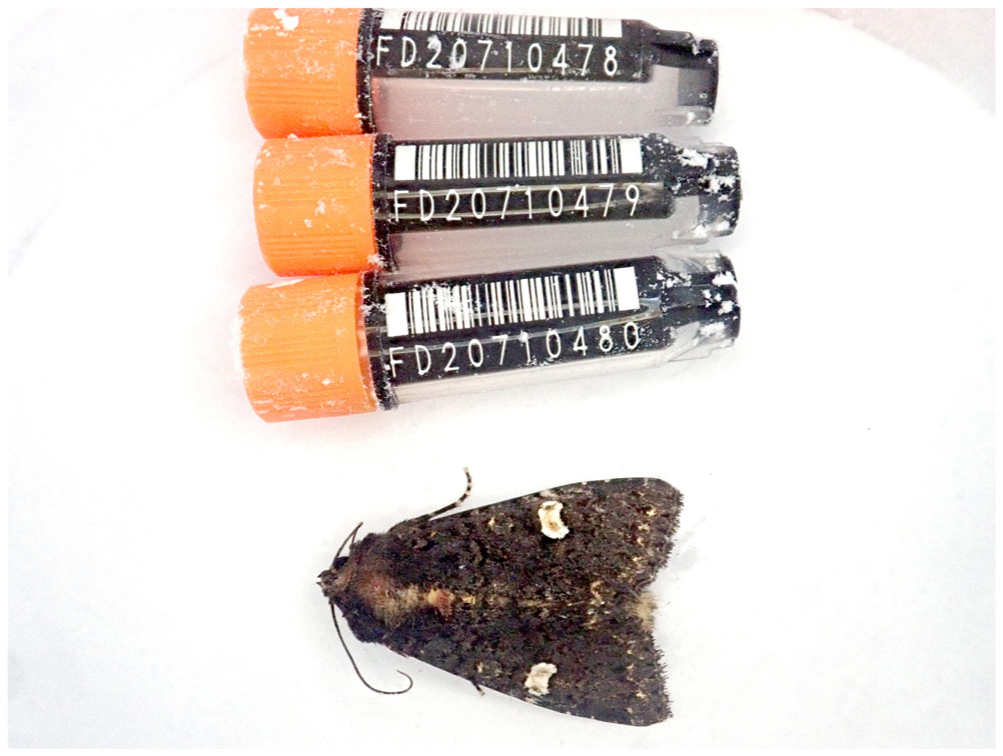
Photograph of the *Melanchra persicariae* (ilMelPers1) specimen used for genome sequencing.

The genome was sequenced from one male *Melanchra persicariae* ( Figure 1) collected from Wytham Woods, Oxfordshire, UK (latitude 51.77, longitude –1.34). A total of 41-fold coverage in Pacific Biosciences single-molecule HiFi long reads was generated. Primary assembly contigs were scaffolded with chromosome conformation Hi-C data. Manual assembly curation corrected three missing joins or mis-joins and removed one haplotypic duplication, reducing the scaffold count by one.

**Figure 2.**
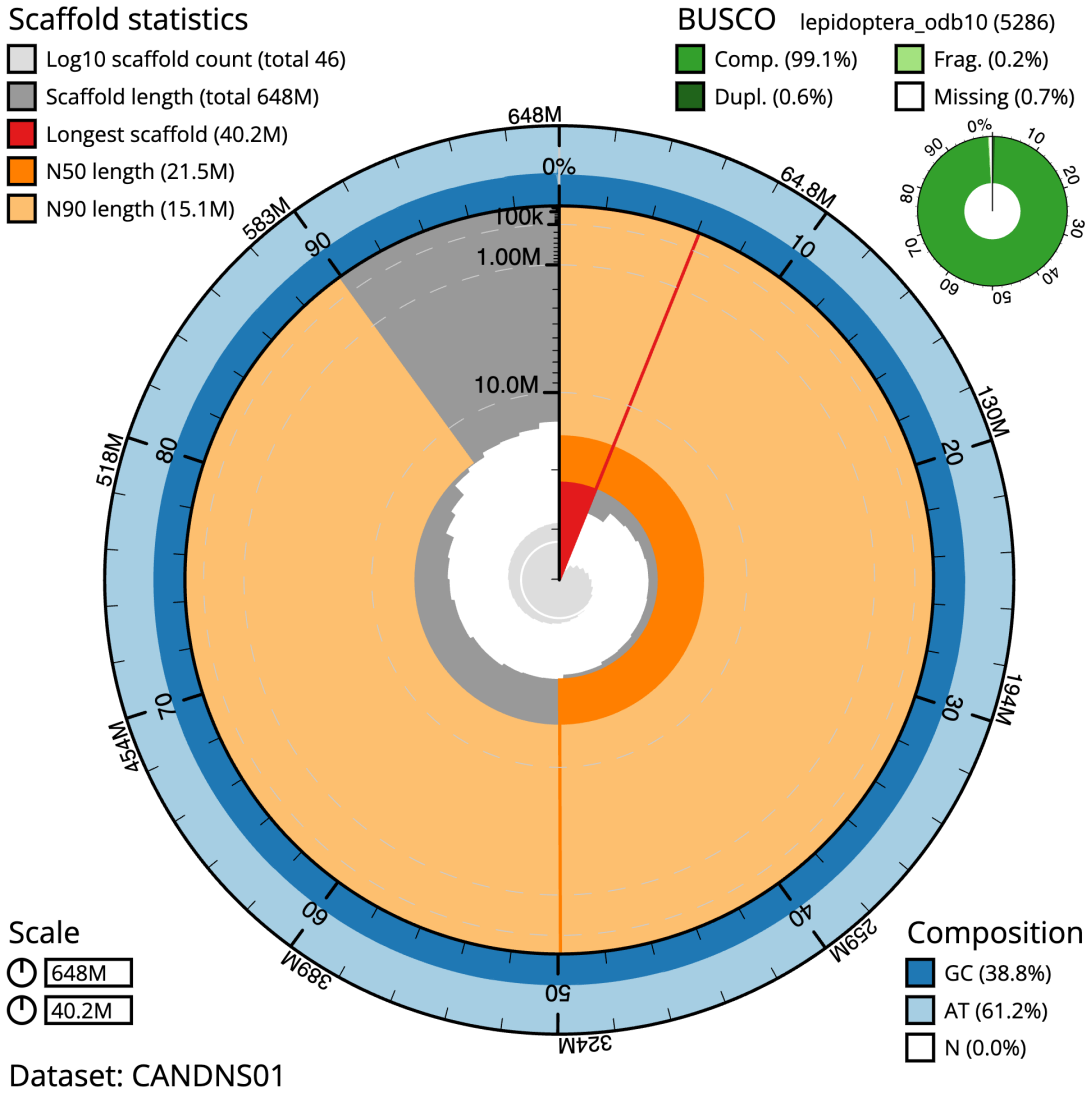
Genome assembly of *Melanchra persicariae*, ilMelPers1.1: metrics. The BlobToolKit Snailplot shows N50 metrics and BUSCO gene completeness. The main plot is divided into 1,000 size-ordered bins around the circumference with each bin representing 0.1% of the 647,911,175 bp assembly. The distribution of scaffold lengths is shown in dark grey with the plot radius scaled to the longest scaffold present in the assembly (40,163,345 bp, shown in red). Orange and pale-orange arcs show the N50 and N90 scaffold lengths (21,548,939 and 15,092,271 bp), respectively. The pale grey spiral shows the cumulative scaffold count on a log scale with white scale lines showing successive orders of magnitude. The blue and pale-blue area around the outside of the plot shows the distribution of GC, AT and N percentages in the same bins as the inner plot. A summary of complete, fragmented, duplicated and missing BUSCO genes in the lepidoptera_odb10 set is shown in the top right. An interactive version of this figure is available at https://blobtoolkit.genomehubs.org/view/ilMelPers1.1/dataset/CANDNS01/snail.

The final assembly has a total length of 647.9 Mb in 46 sequence scaffolds with a scaffold N50 of 21.5 Mb ( Table 1). Most (99.99%) of the assembly sequence was assigned to 31 chromosomal-level scaffolds, representing 30 autosomes and the Z sex chromosome. Chromosome-scale scaffolds confirmed by the Hi-C data are named in order of size ( Figure 2– Figure 5; Table 2). While not fully phased, the assembly deposited is of one haplotype. Contigs corresponding to the second haplotype have also been deposited. The mitochondrial genome was also assembled and can be found as a contig within the multifasta file of the genome submission.

**Table 1.** Genome data for *Melanchra persicariae*, ilMelPers1.1.

Project accession data		
Assembly identifier	ilMelPers1.1	
Species	*Melanchra persicariae*	
Specimen	ilMelPers1	
NCBI taxonomy ID	987979	
BioProject	PRJEB56410	
BioSample ID	SAMEA10978947	
Isolate information	ilMelPers1, male: thorax (genome sequencing); head (Hi-C scaffolding)	
Assembly metrics *		*Benchmark*
Consensus quality (QV)	68.4	*≥ 50*
*k*-mer completeness	100%	*≥ 95%*
BUSCO **	C:99.1%[S:98.5%,D:0.6%], F:0.2%,M:0.7%,n:5286	*C ≥ 95%*
Percentage of assembly mapped to chromosomes	99.99%	*≥ 95%*
Sex chromosomes	Z chromosome	*localised homologous pairs*
Organelles	Mitochondrial genome assembled.	*complete single alleles*
Raw data accessions		
PacificBiosciences SEQUEL II	ERR10499391	
Hi-C Illumina	ERR10313055	
Genome assembly		
Assembly accession	GCA_947386135.1	
*Accession of alternate haplotype*	GCA_947386145.1	
Span (Mb)	647.9	
Number of contigs	108	
Contig N50 length (Mb)	11.6	
Number of scaffolds	46	
Scaffold N50 length (Mb)	21.5	
Longest scaffold (Mb)	40.2	

* Assembly metric benchmarks are adapted from column VGP-2020 of “Table 1: Proposed standards and metrics for defining genome assembly quality” from ( Rhie *et al.*, 2021). ** BUSCO scores based on the lepidoptera_odb10 BUSCO set using v5.3.2. C = complete [S = single copy, D = duplicated], F = fragmented, M = missing, n = number of orthologues in comparison. A full set of BUSCO scores is available at https://blobtoolkit.genomehubs.org/view/ilMelPers1.1/dataset/CANDNS01/busco.

**Figure 3.**
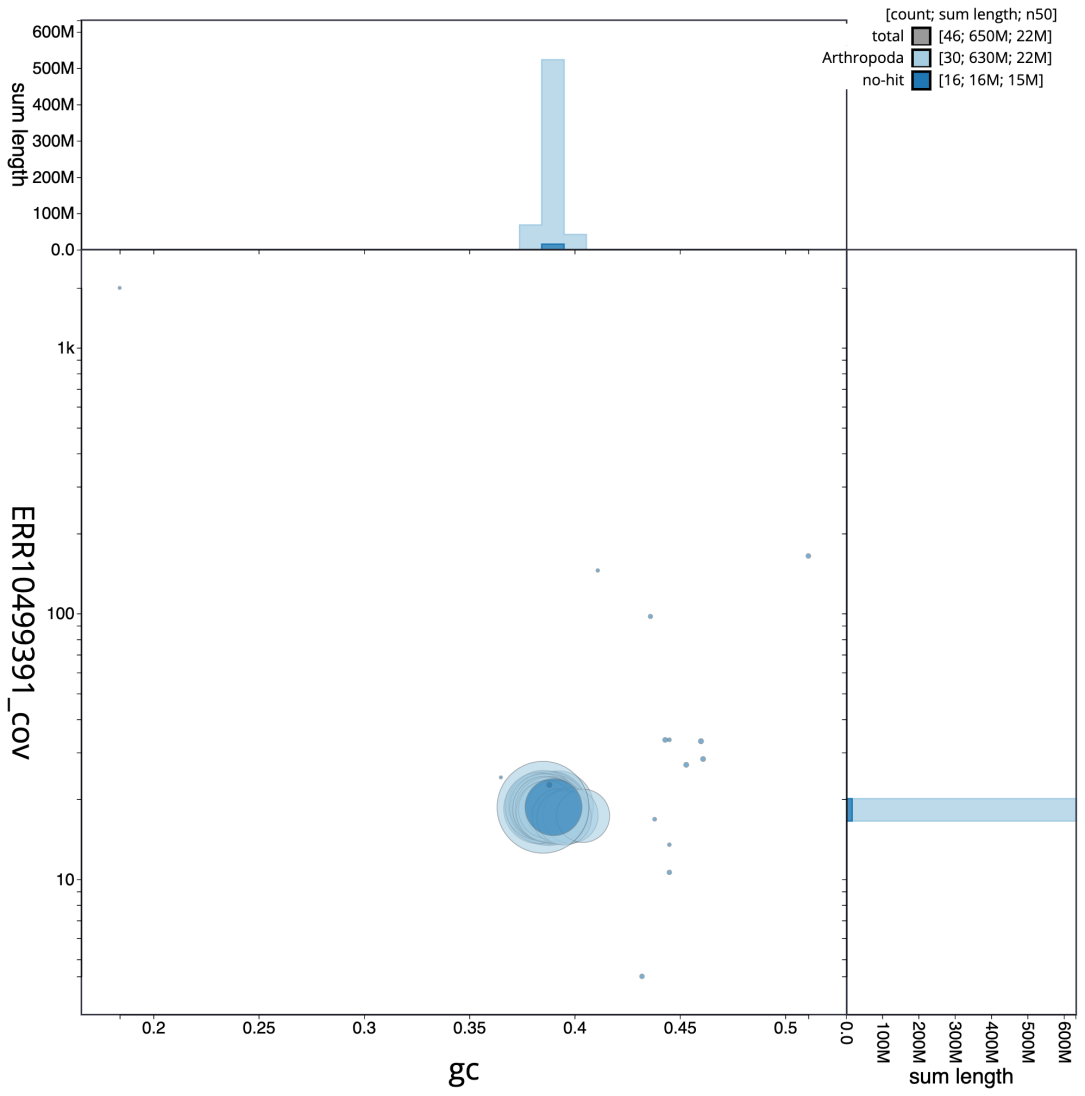
Genome assembly of *Melanchra persicariae*, ilMelPers1.1: BlobToolKit GC-coverage plot. Scaffolds are coloured by phylum. Circles are sized in proportion to scaffold length. Histograms show the distribution of scaffold length sum along each axis. An interactive version of this figure is available at https://blobtoolkit.genomehubs.org/view/ilMelPers1.1/dataset/CANDNS01/blob.

**Figure 4.**
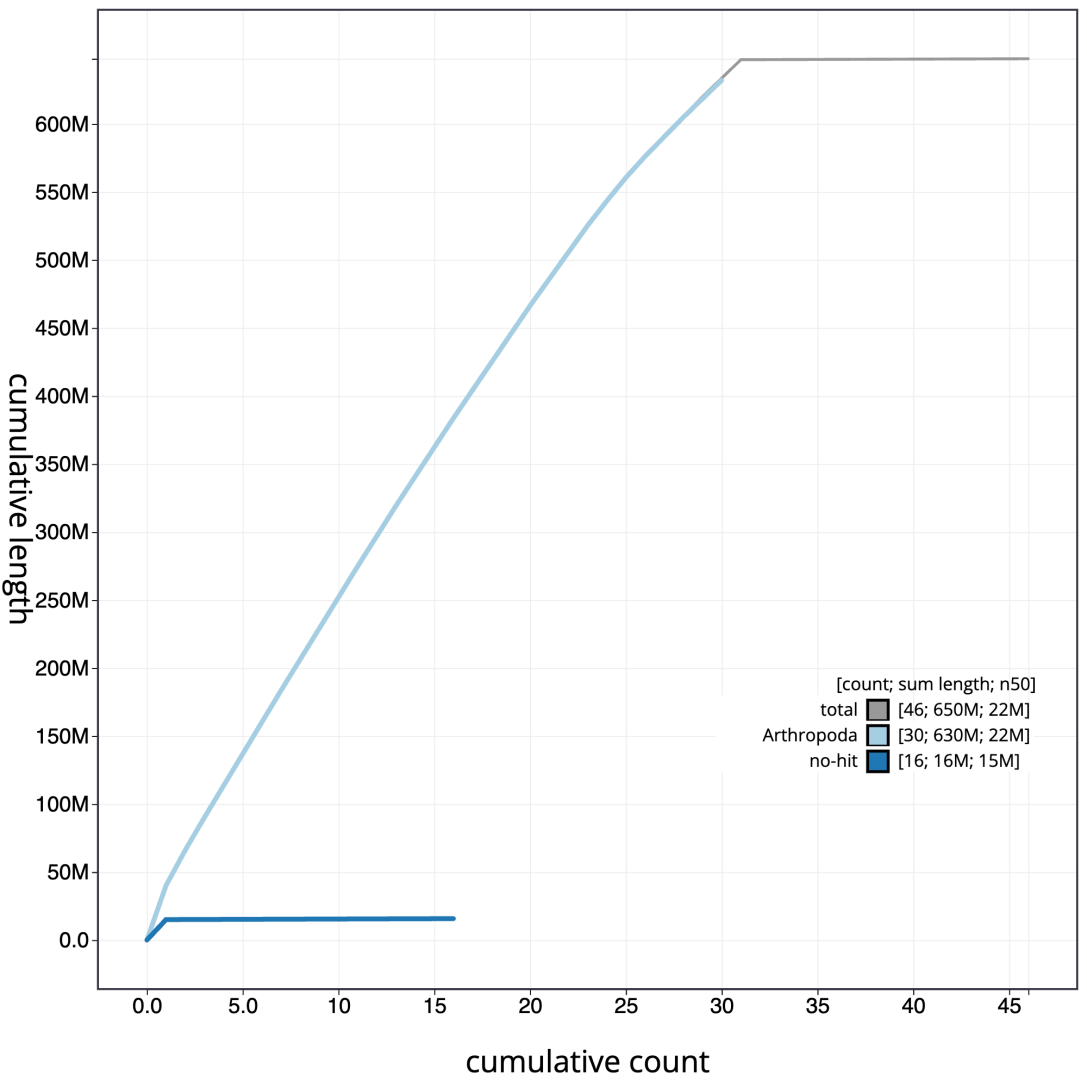
Genome assembly of *Melanchra persicariae*, ilMelPers1.1: BlobToolKit cumulative sequence plot. The grey line shows cumulative length for all scaffolds. Coloured lines show cumulative lengths of scaffolds assigned to each phylum using the buscogenes taxrule. An interactive version of this figure is available at https://blobtoolkit.genomehubs.org/view/ilMelPers1.1/dataset/CANDNS01/cumulative.

**Figure 5.**
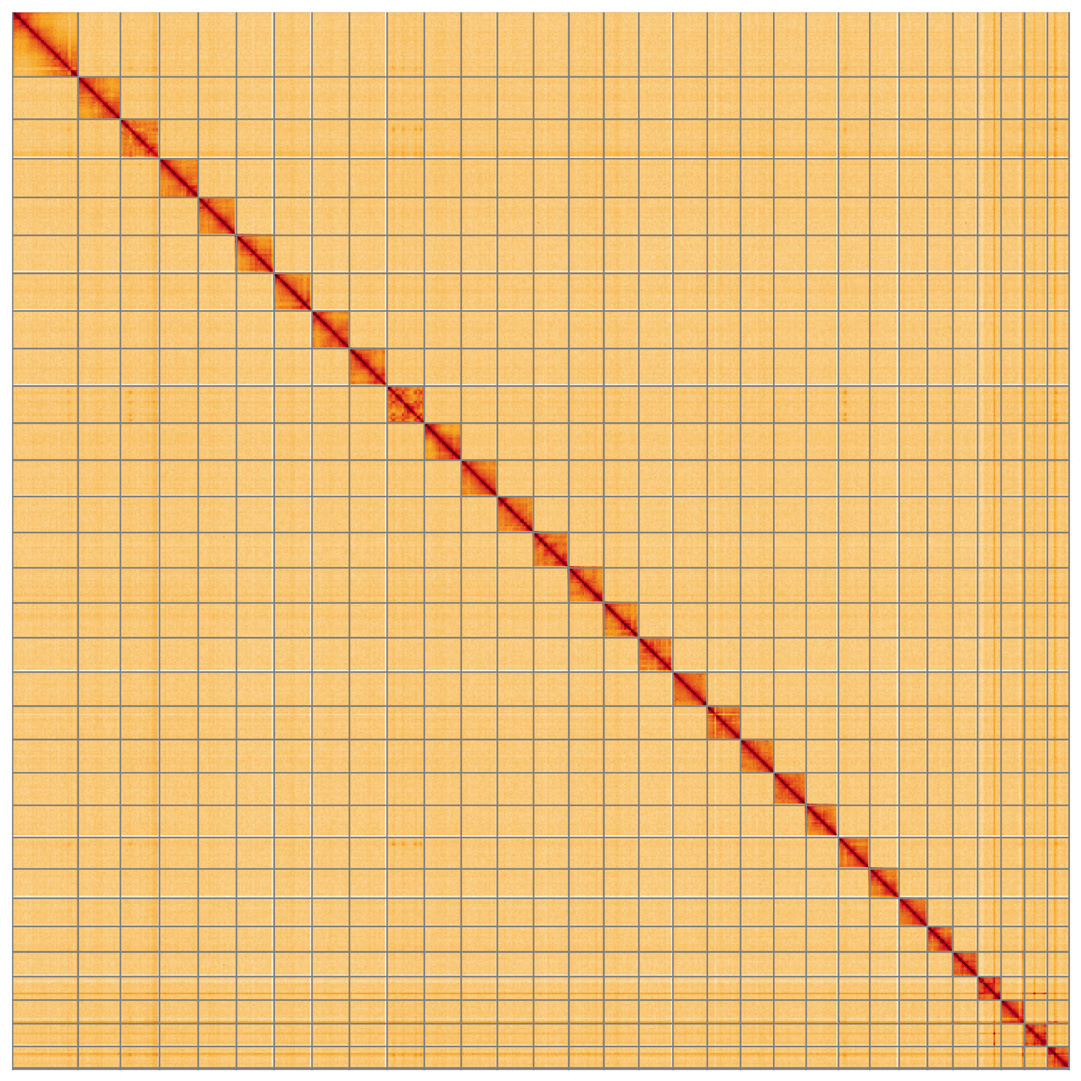
Genome assembly of *Melanchra persicariae*, ilMelPers1.1: Hi-C contact map of the ilMelPers1.1 assembly, visualised using HiGlass. Chromosomes are shown in order of size from left to right and top to bottom. An interactive version of this figure may be viewed at https://genome-note-higlass.tol.sanger.ac.uk/l/?d=DpyT6iq2Rr2JBQ2hDL_f8g.

**Table 2.** Chromosomal pseudomolecules in the genome assembly of *Melanchra persicariae*, ilMelPers1.

INSDC accession	Chromosome	Size (Mb)	GC%
OX376644.1	1	25.93	38.8
OX376645.1	2	24.03	39.2
OX376646.1	3	23.74	38.9
OX376647.1	4	23.35	38.6
OX376648.1	5	23.16	38.3
OX376649.1	6	23.12	38.7
OX376650.1	7	23	38.4
OX376651.1	8	22.96	38.5
OX376652.1	9	22.74	38.7
OX376653.1	10	22.63	38.8
OX376654.1	11	22.25	39
OX376655.1	12	22.22	38.5
OX376656.1	13	21.55	38.3
OX376657.1	14	21.51	38.6
OX376658.1	15	21.36	38.7
OX376659.1	16	20.95	38.5
OX376660.1	17	20.92	38.9
OX376661.1	18	20.42	39
OX376662.1	19	20.39	38.8
OX376663.1	20	19.92	38.6
OX376664.1	21	19.63	38.6
OX376665.1	22	19.32	38.7
OX376666.1	23	18.04	38.7
OX376667.1	24	17.27	39.1
OX376668.1	25	15.54	38.7
OX376669.1	26	15.09	38.9
OX376670.1	27	14.49	39.3
OX376671.1	28	14.35	39.8
OX376672.1	29	13.95	39.5
OX376673.1	30	13.23	40.4
OX376643.1	Z	40.16	38.5
OX376674.1	MT	0.02	18.7

The estimated Quality Value (QV) of the final assembly is 68.4 with *k*-mer completeness of 100%, and the assembly has a BUSCO v5.3.2 completeness of 99.1% (single = 98.5%, duplicated = 0.6%), using the lepidoptera_odb10 reference set ( *n* = 5,286).

Metadata for specimens, spectral estimates, sequencing runs, contaminants and pre-curation assembly statistics can be found at https://links.tol.sanger.ac.uk/species/987979.

## Methods

### Sample acquisition and nucleic acid extraction

A male *Melanchra persicariae* specimen (individual ilMelPers1, specimen Ox001680) was collected from Wytham Woods, Oxfordshire (biological vice-county: Berkshire), UK (latitude 51.77, longitude –1.34) on 17 July 2021. The specimen was caught using a light trap in woodland habitat by Douglas Boyes (University of Oxford). The specimen was identified by the collector and then snap-frozen on dry ice.

DNA was extracted at the Tree of Life laboratory, Wellcome Sanger Institute (WSI). The ilMelPers1 sample was weighed and dissected on dry ice with head tissue set aside for Hi-C sequencing. Thorax tissue was disrupted using a Nippi Powermasher fitted with a BioMasher pestle. High molecular weight (HMW) DNA was extracted using the Qiagen MagAttract HMW DNA extraction kit. HMW DNA was sheared into an average fragment size of 12–20 kb in a Megaruptor 3 system with speed setting 30. Sheared DNA was purified by solid-phase reversible immobilisation using AMPure PB beads with a 1.8X ratio of beads to sample to remove the shorter fragments and concentrate the DNA sample. The concentration of the sheared and purified DNA was assessed using a Nanodrop spectrophotometer and Qubit Fluorometer and Qubit dsDNA High Sensitivity Assay kit. Fragment size distribution was evaluated by running the sample on the FemtoPulse system.

### Sequencing

Pacific Biosciences HiFi circular consensus DNA sequencing libraries were constructed according to the manufacturers’ instructions. DNA sequencing was performed by the Scientific Operations core at the WSI on Pacific Biosciences SEQUEL II (HiFi) instrument. Hi-C data were also generated from head tissue of ilMelPers1 using the Arima2 kit and sequenced on the Illumina NovaSeq 6000 instrument.

### Genome assembly, curation and evaluation

Assembly was carried out with Hifiasm ( Cheng *et al.*, 2021) and haplotypic duplication was identified and removed with purge_dups ( Guan *et al.*, 2020). The assembly was then scaffolded with Hi-C data ( Rao *et al.*, 2014) using YaHS ( Zhou *et al.*, 2023). The assembly was checked for contamination as described previously ( Howe *et al.*, 2021). Manual curation was performed using HiGlass ( Kerpedjiev *et al.*, 2018) and Pretext ( Harry, 2022). The mitochondrial genome was assembled using MitoHiFi ( Uliano-Silva *et al.*, 2022), which runs MitoFinder ( Allio *et al.*, 2020) or MITOS ( Bernt *et al.*, 2013) and uses these annotations to select the final mitochondrial contig and to ensure the general quality of the sequence. To evaluate the assembly, MerquryFK was used to estimate consensus quality (QV) scores and *k*-mer completeness ( Rhie *et al.*, 2020). The genome was analysed within the BlobToolKit environment ( Challis *et al.*, 2020) and BUSCO scores ( Manni *et al.*, 2021; Simão *et al.*, 2015) were calculated. Table 3 contains a list of software tool versions and sources.

**Table 3.** Software tools: versions and sources.

Software tool	Version	Source
BlobToolKit	4.0.7	https://github.com/blobtoolkit/blobtoolkit
BUSCO	5.3.2	https://gitlab.com/ezlab/busco
Hifiasm	0.16.1-r375	https://github.com/chhylp123/hifiasm
HiGlass	1.11.6	https://github.com/higlass/higlass
Merqury	MerquryFK	https://github.com/thegenemyers/MERQURY.FK
MitoHiFi	2	https://github.com/marcelauliano/MitoHiFi
PretextView	0.2	https://github.com/wtsi-hpag/PretextView
purge_dups	1.2.3	https://github.com/dfguan/purge_dups
YaHS	yahs-1.1.91eebc2	https://github.com/c-zhou/yahs

### Ethics and compliance issues

The materials that have contributed to this genome note have been supplied by a Darwin Tree of Life Partner. The submission of materials by a Darwin Tree of Life Partner is subject to the Darwin Tree of Life Project Sampling Code of Practice. By agreeing with and signing up to the Sampling Code of Practice, the Darwin Tree of Life Partner agrees they will meet the legal and ethical requirements and standards set out within this document in respect of all samples acquired for, and supplied to, the Darwin Tree of Life Project. All efforts are undertaken to minimise the suffering of animals used for sequencing. Each transfer of samples is further undertaken according to a Research Collaboration Agreement or Material Transfer Agreement entered into by the Darwin Tree of Life Partner, Genome Research Limited (operating as the Wellcome Sanger Institute), and in some circumstances other Darwin Tree of Life collaborators.

## Data Availability

European Nucleotide Archive: *Melanchra persicariae* (dot moth). Accession number PRJEB56410; https://identifiers.org/ena.embl/PRJEB56410. ( Wellcome Sanger Institute, 2022) The genome sequence is released openly for reuse. The *Melanchra persicariae* genome sequencing initiative is part of the Darwin Tree of Life (DToL) project. All raw sequence data and the assembly have been deposited in INSDC databases. The genome will be annotated using available RNA-Seq data and presented through the Ensembl pipeline at the European Bioinformatics Institute. Raw data and assembly accession identifiers are reported in Table 1.

## Author information

Members of the University of Oxford and Wytham Woods Genome Acquisition Lab are listed here: https://doi.org/10.5281/zenodo.4789928.

Members of the Darwin Tree of Life Barcoding collective are listed here: https://doi.org/10.5281/zenodo.4893703.

Members of the Wellcome Sanger Institute Tree of Life programme are listed here: https://doi.org/10.5281/zenodo.4783585.

Members of Wellcome Sanger Institute Scientific Operations: DNA Pipelines collective are listed here: https://doi.org/10.5281/zenodo.4790455.

Members of the Tree of Life Core Informatics collective are listed here: https://doi.org/10.5281/zenodo.5013541.

Members of the Darwin Tree of Life Consortium are listed here: https://doi.org/10.5281/zenodo.4783558.
