# Supplementary material for: The genome sequence of the Dot Moth, Melanchra persicariae (Linnaeus, 1761)
Source: Wellcome Open Res. 2023 Apr 27;8:190. [Version 1] doi: 10.12688/wellcomeopenres.19410.1 (PMC12624260; doi:10.12688/wellcomeopenres.19410.1)
Supplement: Peer review history

# Reviewer report: Reviewer response for version 1

**DOI:** 10.21956/wellcomeopenres.21500.r136053

**Published:** 2025-11-17

**Authors:** Bhagya C. Thimmappa

**Affiliations:** 1 Universite de Montreal, Montreal, Québec, Canada; 2 USDA-ARS Beltsville Agricultural Research Center, Beltsville, Maryland, USA

In the article titled “The genome sequence of the Dot Moth, *Melanchra persicariae* (Linnaeus, 1761)”, the authors report the first nuclear and mitochondrial genome assemblies for the male *Melanchra persicariae*. The reported nuclear genome assembly is 647.9 megabases in size, and the mitochondrial genome assembly is of 15.4 kilobases in length. The high-quality assembly was achieved using Pacific Biosciences single-molecule HiFi long reads and Hi-C data. Further manual curation resulted in a high-quality assembly as evidenced by the 99.1% BUSCO value. The methods are described in sufficient detail, and the data have been made publicly available.

 Minor comments:

1. In Table 1, please provide a hyperlink to the assembly accession number for easy access.
2. Some numbers in Table 1 and the NCBI accession do not match; please check. For instance, the number of contigs and, number of scaffolds.
3. Which other polyphagy moths are closely related to *Melanchra persicariae*?

Are sufficient details of methods and materials provided to allow replication by others?

Yes

Is the rationale for creating the dataset(s) clearly described?

Yes

Are the datasets clearly presented in a useable and accessible format?

Yes

Are the protocols appropriate and is the work technically sound?

Yes

Reviewer Expertise:

Bioinformatics, multi-omics, mycology, plant-microbe interactions

I confirm that I have read this submission and believe that I have an appropriate level of expertise to confirm that it is of an acceptable scientific standard.

# Reviewer report: Reviewer response for version 1

**DOI:** 10.21956/wellcomeopenres.21500.r135006

**Published:** 2025-11-17

**Authors:** Bin Zhang

**Affiliations:** 1 Qingdao Agricultural University, Shenzhen, China

This data note presents a high-quality chromosome-level genome assembly of *Melanchra persicariae* (Dot Moth), a member of the ecologically important family Noctuidae, as part of the Darwin Tree of Life Project. The assembly meets the Vertebrate Genomes Project (VGP) 2020 standards (e.g., consensus QV ≥50, BUSCO completeness ≥95%) and provides comprehensive details on sequencing, assembly, and curation workflows. By integrating PacBio HiFi long reads and Hi-C data, the authors successfully scaffolded 99.99% of the genome into 31 chromosomal pseudomolecules (including the Z sex chromosome) and assembled the mitochondrial genome. This work fills a critical gap in genomic resources for Noctuidae, supporting future research on lepidopteran phylogenetics, polyphagous adaptation, and conservation genomics. However, several issues related to sample representativeness, genomic detail, and scientific context need clarification to maximize the manuscript’s utility.

 1. The assembly is derived from a single male specimen collected from Wytham Woods, UK. M. persicariae has a broad distribution (Europe to Japan) and exhibits population declines in parts of its range, yet the manuscript does not discuss whether this individual captures the species’ genetic diversity (e.g., population-specific variants, heterozygosity differences across ranges). This oversight limits the assembly’s utility as a “reference genome” for the entire species.

 2. biguous sex chromosome characterization: Only the Z sex chromosome is reported, with no explicit explanation of the species’ sex determination system. In Lepidoptera, males typically have a ZZ system (females are ZW), meaning the male specimen should not possess a W chromosome. However, the manuscript fails to state this biological fact, leading to confusion about whether the W chromosome was missed or is naturally absent.

 3. While the mitochondrial genome length (15.4 kb) is noted, key evolutionary features are missing: (1) gene order (e.g., arrangement of protein-coding genes, rRNA, tRNA); (2) GC content of functional regions (e.g., control region vs. coding regions); (3) comparisons with mitochondrial genomes of congeneric (e.g., other Melanchra species) or confamilial (e.g., Spodoptera frugiperda) species. This reduces its value for studies of Noctuidae mitochondrial evolution.

 4. The manuscript states the genome “will be annotated using available RNA-Seq data” but provides no preliminary results (e.g., number of predicted protein-coding genes, functional enrichment of detoxification genes) or timelines for release. This prevents linking the assembly to M. persicariae’s polyphagous adaptation (e.g., genes for detoxifying plant secondary compounds).

 5. The assembly offers opportunities to resolve Noctuidae phylogeny or understand polyphagy, but the manuscript does not discuss such applications. For example, it does not compare chromosomal synteny with other Noctuidae species or highlight genes potentially involved in hostplant range expansion.

 The manuscript presents a scientifically valuable, high-quality genome assembly that addresses a critical gap in Noctuidae genomics. The identified issues are primarily related to contextualization and technical clarifications, not fundamental flaws in the assembly. **Minor Revision** is recommended; the revised version should resolve the above concerns to ensure the assembly maximizes its impact for lepidopteran research.

Are sufficient details of methods and materials provided to allow replication by others?

Yes

Is the rationale for creating the dataset(s) clearly described?

Yes

Are the datasets clearly presented in a useable and accessible format?

Yes

Are the protocols appropriate and is the work technically sound?

Yes

Reviewer Expertise:

Entomology and Bioinformatics

I confirm that I have read this submission and believe that I have an appropriate level of expertise to confirm that it is of an acceptable scientific standard.

# Reviewer report: Reviewer response for version 1

**DOI:** 10.21956/wellcomeopenres.21500.r136048

**Published:** 2025-10-31

**Authors:** Arun Arumugaperumal

**Affiliations:** 1 Rajalakshmi Engineering College, Chennai, Tamil Nadu, India

The authors have presented the whole genome of *Melanchra persicariae*, also known as the Dot Moth. Long-read sequencing and Hi-C mapping data have been used to arrive at a high-quality genome assembly. The assembly has a size of 647.9 Mb spread among 31 chromosome molecules. The authors have used a male specimen and thus they have captured the Z sex chromosome. They could have used a female specimen to obtain the W sex chromosome. The mitogenome of size 15.4 kb has also been reported. The contig N50 value is well above the benchmark value. BUSCO completeness of 99.1 % indicates that the genome is complete with respect to the sequences in the database. The annotation of the genome has not been released by Ensembl during the time of review. The article can be indexed.

Are sufficient details of methods and materials provided to allow replication by others?

Yes

Is the rationale for creating the dataset(s) clearly described?

Yes

Are the datasets clearly presented in a useable and accessible format?

Yes

Are the protocols appropriate and is the work technically sound?

Yes

Reviewer Expertise:

Bioinformatics; Genomics

I confirm that I have read this submission and believe that I have an appropriate level of expertise to confirm that it is of an acceptable scientific standard.
